# A systematic review and meta-analysis of risk factors for reoperation after degenerative lumbar spondylolisthesis surgery

**DOI:** 10.1186/s12893-023-02082-8

**Published:** 2023-07-05

**Authors:** Yuzhou Chen, Yi Zhou, Junlong Chen, Yiping Luo, Yongtao Wang, Xiaohong Fan

**Affiliations:** 1 Chengdu University of Traditional Chinese Medicine, Chengdu, 610075 P.R. China; 2 Department of Orthopedics, Hospital of Chengdu University of Traditional Chinese Medicine, No.39 Shi-Er-Qiao Road, Jinniu District, Chengdu, 610075 P.R. China; 3 Department of Traditional Chinese Medicine, The Traditional Chinese Medicine Hospital of Wenjiang District, Chengdu, 611130 P.R. China; 4 Department of Anorectal, The Traditional Chinese Medicine Hospital of Wenjiang District, Chengdu, 611130 P.R. China; 5 Department of Gynecology, Hospital of Chengdu University of Traditional Chinese Medicine, Chengdu, 610075 P.R. China

**Keywords:** Degenerative lumbar spondylolisthesis, Reoperation, Risk factors, Meta-analysis

## Abstract

**Background:**

Considering the high reoperation rate in degenerative lumbar spondylolisthesis (DLS) patients undergoing lumbar surgeries and controversial results on the risk factors for the reoperation, we performed a systematic review and meta-analysis to explore the reoperation rate and risk factors for the reoperation in DLS patients undergoing lumbar surgeries.

**Methods:**

Literature search was conducted from inception to October 28, 2022 in Pubmed, Embase, Cochrane Library, and Web of Science. Odds ratio (OR) was used as the effect index for the categorical data, and effect size was expressed as 95% confidence interval (CI). Heterogeneity test was performed for each outcome effect size, and subgroup analysis was performed based on study design, patients, surgery types, follow-up time, and quality of studies to explore the source of heterogeneity. Results of all outcomes were examined by sensitivity analysis. Publication bias was assessed using Begg test, and adjusted using trim-and-fill analysis.

**Results:**

A total of 39 cohort studies (27 retrospective cohort studies and 12 prospective cohort studies) were finally included in this systematic review and meta-analysis. The overall results showed a 10% (95%CI: 8%-12%) of reoperation rate in DLS patients undergoing lumbar surgeries. In surgery types subgroup, the reoperation rate was 11% (95%CI: 9%-13%) for decompression, 10% (95%CI: 7%-12%) for fusion, and 9% (95%CI: 5%-13%) for decompression and fusion. An increased risk of reoperation was found in patients with obesity (OR = 1.91, 95%CI: 1.04–3.51), diabetes (OR = 2.01, 95%CI: 1.43–2.82), and smoking (OR = 1.51, 95%CI: 1.23–1.84).

**Conclusions:**

We found a 10% of reoperation rate in DLS patients after lumbar surgeries. Obesity, diabetes, and smoking were risk factors for the reoperation.

**Supplementary Information:**

The online version contains supplementary material available at 10.1186/s12893-023-02082-8.

## Background

Degenerative lumbar spondylolisthesis (DLS) refers to anterolisthesis of one vertebral body over another vertebral body secondary to osteoarthritic degeneration, leading to spinal canal stenosis [1]. DLS is an aging-related disease, and its incidence is increasing under the background of the global population aging [2]. Each year, 39 million individuals are diagnosed with DLS, accounting for a global prevalence of 0.53% [3]. DLS may be accompanied with low back pain, radiculopathy, or neurogenic claudication [2].

Surgeries have been regarded as the standard treatment modality for intractable cases [4]. The proportion of lumbar surgeries increases by more than two-fold not only because of elevated prevalence of degenerative lumbar spine disease but also because of improved surgical techniques, good outcomes, and increased hospitals and surgeons [5, 6]. However, due to complications (such as fusion failure, persistent pain, and infection), progressive degenerative changes-related diseases, or an unrelated previous surgeries, some patients require reoperation [7]. Despite improvements in surgical skills and techniques, the reoperation rate is still unimproved, with a 10-year reoperation rate of about 15% [7]. Given the high prevalence and chronicity of DLS, understanding the risk factors for reoperation is important [8]. Park et al. have revealed the longitudinal trends in the lumbar reoperation rate, and the reoperation was associated with demographics, comorbidities, primary surgery type, and preoperative spinal pathology [9]. Noh et al. haven found lifestyle-related factors, such as smoking, drinking, and exercise, were associated with the higher rate of reoperation [10]. However, results of studies on the risk factors for reoperation of DLS patients remain controversial. Rabah et al., have reported that diabetes was related to greater risk of reoperation [11], while Khan et al. reported no significant association between diabetes and reoperation [12]. In the study performed by Zhong et al., obesity was found to be associated with a higher incidence of unplanned reoperations [8]. Nevertheless, Kuo et al. found that obesity was not significantly associated with the reoperation [13].

Considering the controversial results, we aimed to perform a systematic review and meta-analysis to evaluate the incidence and risk factors of reoperation in DLS patients for the purpose of improving surgical outcomes and prognosis.

## Methods

### Literature search strategy

This study was performed based on the Preferred Reporting Items for Systematic Reviews and Meta-Analyses (PRISMA) [14]. Two researchers (YZC and YZ) conducted the literature search from September 28, 2022 to October 28, 2022 in Pubmed, Embase, Cochrane Library, and Web of Science. Consensus was reached by discussion; if consensus cannot be reached by discussion, a third researcher (XHF) was consulted. Search terms were “degenerative spinal diseases” OR “degenerative spondylolisthesis” OR “degenerative lumbar spondylolisthesis” OR “degenerative cervical spondylolisthesis” AND “spinal surgery” OR “fusion” OR “reoperation” OR “repeat surgery” OR “risk factor”.

### Selection criteria

Studies meeting the following inclusion criteria were selected: (1) population: DLS patients; (2) patients undergoing lumbar surgeries, including decompression surgeries and fusion surgeries; (3) outcome: reoperation rate and risk factors; and (4) studies: cohort studies. The population included DLS patients and DLS patients with lumbar spinal stenosis (LSS). Fusion surgeries included posterolateral lumbar fusion (PLF) and lumbar interbody fusion (LIF). Reoperation was defined as the secondary lumbar surgeries due to progression of lumbar degenerative changes or postoperative instability [8]. Risk factors included body mass index (BMI), sex, age, diabetes, smoking, and more bleeding.

Studies were excluded by meeting one of the following criteria: (1) animal studies; (2) other degenerative spinal diseases including lumbar spine stenosis, degenerative disc disease, and degenerative cervical spondylosis; (3) not English articles; (4) unable to extract data; (5) case reports, conference abstracts, letters, reviews, and meta-analysis.

### Data extraction and critical appraisal

The following data were extracted: the first author, year of publication, country, study design, patients, definition of spondylolisthesis, sample size, age, sex, BMI, disease duration, surgery types, follow-up time, number of reoperations, reasons for reoperation, reoperation methods, and risk factors of reoperation. The Newcastle–Ottawa Scale (NOS) was applied to evaluate the quality of cohort studies [15]. This scale consisted of three items: selection, comparability, and outcome. This scale was scored a total of 9 points, and divided into low quality (0–3 points), fair quality (4–6 points), and high quality (7–9 points) [15].

### Statistical analysis

All statistical analyses were performed using Stata15.1 software (Stata Corporation, College Station, TX, USA). Rate was used as the effect index in the analysis of reoperation rate. Odds ratio (OR) was used as the effect index for categorical data, and effect size was represented as 95% confidence interval (CI). Heterogeneity test was performed for each outcome effect size, and results were quantified as I-squared (I^2^). Random-effect model was used for analysis if I^2^ ≥ 50%, and fixed-effect model was used if I^2^ < 50%. For the high heterogeneity (I^2^ ≥ 50%), subgroup analysis was conducted based on study design, patients, surgery types, follow-up time, and quality of studies to explore the source of heterogeneity. Sensitivity analysis was carried out for all outcomes. Begg test was used to assess publication bias for the outcome included more than 10 articles. Trim-and-fill analysis was used to adjust the publication bias. *P* < 0.05 was considered statistically significant.

## Results

### Identification of studies and characteristics of patients

**Fig. 1:**
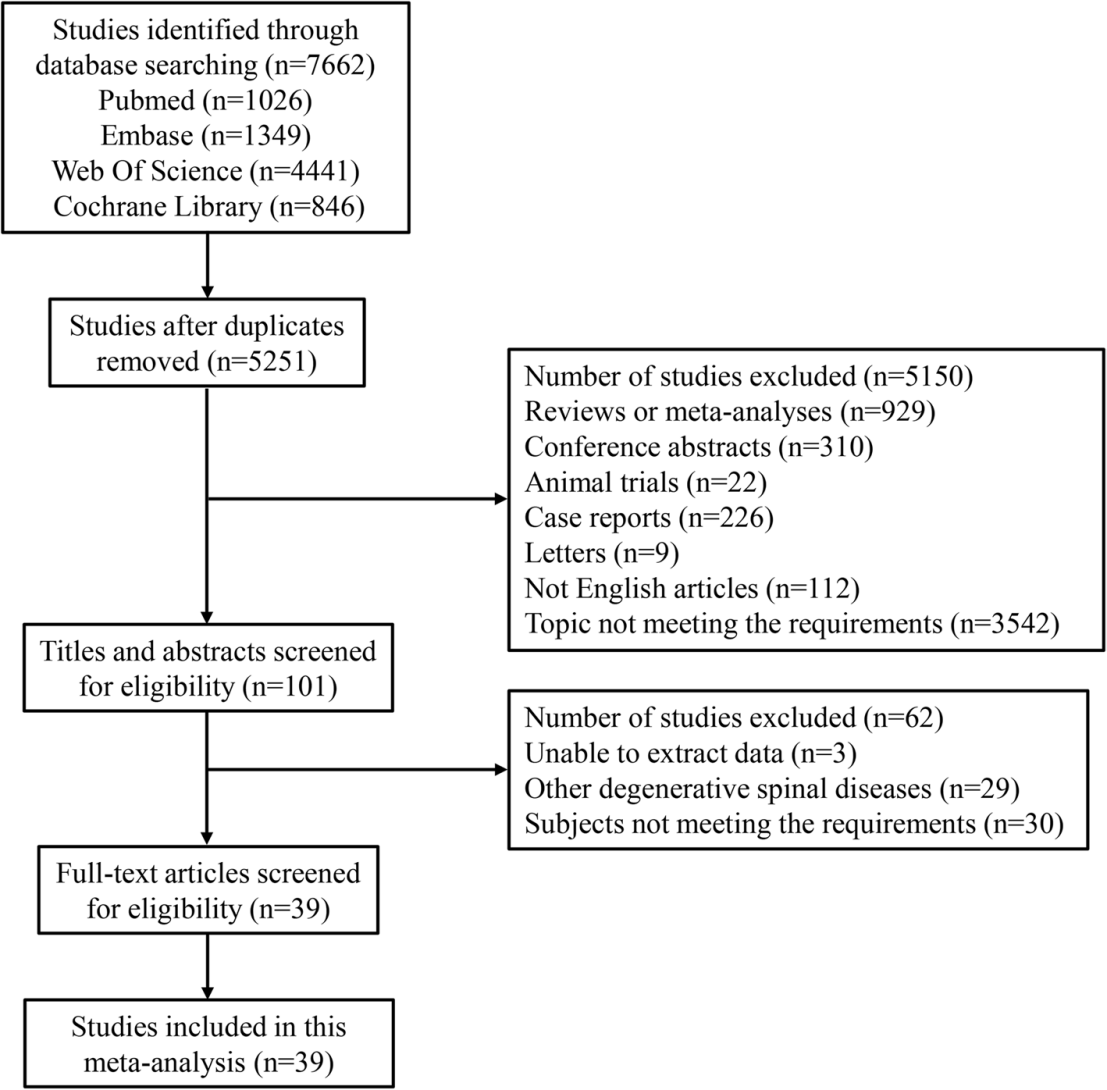
Flowchart of identifying studies

A total of 7,662 articles were searched from Pubmed (*n* = 1026), Embase (*n* = 1349), Web of Science (*n* = 4441), and Cochrane Library (*n* = 846). After removing the duplicates, 5,251 articles remained. Further, 5,150 articles were excluded due to publishing as reviews or meta-analyses (*n* = 929), conference abstracts (*n* = 310), animal trials (*n* = 22), case reports (*n* = 226), and letters (*n* = 9), not English articles (*n* = 112), and topic not meeting the requirements (*n* = 3542). In the remaining 101 articles, we further excluded 3 articles unable to extract data, 29 articles reporting other degenerative spinal diseases, and 30 articles with topic not meeting the requirements. Finally, 39 cohort studies were retained in this meta-analysis [7, 8, 10–13, 16–48], with 27 retrospective cohort studies and 12 prospective cohort studies. The flow diagram of our searching was displayed in Fig. 1. In the included studies, 28 studies were assessed as fair quality, and 11 studies were assessed as high quality. Characteristics of the included studies were presented in Table 1.

**Table 1:** Characteristics of included patients

Author	Year	Country	Study design	Patients	Definition of spondylolisthesis	Sample size	Age (years)	Male/female
Salimi	2022	Japan	Retrospective cohort	DLS with LSS	≥ 3 mm anterior slippage	50	69.4 ± 9.5	23/27
Noh	2022	Korea	Retrospective cohort	DLS with or without LSS	NA	DLS with LSS: 3840; DLS without LSS: 255	60.5 ± 9.1	NA
Moayeri	2022	Canada	Retrospective cohort	DLS with LSS	grade 1	140	68.0 ± 10.1	64/76
Liang	2022	China	Retrospective cohort	DLS with LSS	grade 1	104 (PMTD: 53; MIS TLIF: 51)	62.06 ± 13.6; 59.94 ± 8.3	27/26; 17/34
Joelson	2022	Sweden	Prospective cohort	DLS with LSS	slip > 3 mm	372 (decompression and fusion: 228; decompression only: 144)	63.9 ± 8.9; 69.4 ± 9.4	79/149; 47/97
Georgiou	2022	USA	Retrospective cohort	DLS	single-level DLS	51619 (anterior fusion: 14971; posterior fusion: 36648)	≥ 75: 1229; 2514	5629/9342; 12904/23744
Chan	2021	USA	Retrospective cohort	DLS	NA	15658	62.5	5762/9896
Takaoka	2021	Japan	Retrospective cohort	DLS with LSS	NA	145 (OLIF: 66; TLIF: 79)	66 ± 12; 71 ± 9	28/38; 37/42
Sugiura	2021	Japan	Retrospective cohort	DLS	slippage at L3 or L4 of > 3%	202 (BPL: 51; PLIF: 106)	70.4 ± 8.4;69.1 ± 7.7	21/30; 38/68
Rabah	2021	USA	Retrospective cohort	DLS	NA	6260	60.27 ± 12.49	2341/3919
Mimura	2021	Japan	Retrospective cohort	DLS	grade 1 and 2	41	69.8 ± 7.1	19/22
Katuch	2021	Slovakia	Prospective cohort	DLS	NA	333 (TLIF: 119; PLIF:214)	55.21 ± 9.22; 56.51 ± 10.71	52/67; 95/119
Joelson	2021	Sweden	Prospective cohort	DLS with LSS	slip > 3 mm on preoperative radiographs	1935 (Decompression and fusion: 1338; decompression only: 597)	65 ± 9.1; 69 ± 9.9	NA
Badhiwala	2021	Canada	Retrospective cohort	DLS	NA	1804 (laminectomy alone: 802; laminectomy plus fusion: 1002)	64.4 ± 11.6; 62.7 ± 12.1	305/497; 348/654
Zhong	2020	China	Retrospective cohort	DLS	one-level or two-level DLS	1100 (posterolateral fusion: 650; intervertebral fusion: 450)	72.80 ± 11.7; 71.90 ± 10.7	NA
Nyström	2020	Sweden	Retrospective cohort	DLS	slip > 3 mm	200	64.8	92/108
Lee	2020	Korea	Prospective cohort	DLS	NA	620 (decompressive laminectomy: 383; PLIF: 171; ALIF: 66)	NA	NA
Khan	2020	USA	Retrospective cohort	DLS	grade 1 and 2	850	58.35 ± 13.78	397/453
Khan	2019	USA	Retrospective cohort	DLS	grade 1 and 2	141	62.28 ± 10.7	65/76
Karsy	2020	USA	Prospective cohort	DLS	grade 1	608	< 60: 239; 60–70: 209; 71–80: 128; > 80: 32	350/258
Chan	2020	USA	Prospective cohort	DLS	grade 1	297 (MIS-TLIF: 72; Open TLIF: 225)	62.1 ± 10.6; 59.5 ± 11.7	32/40; 82/143
Bisson	2020	USA	Retrospective cohort	DLS	grade 1	140 (MIS decompression: 71; Open decompression: 69)	72.264 ± 9.662; 66.913 ± 12.578	74/66; 32/39
Minamide	2019	USA	Prospective cohort	DLS with LSS	single-level DLS at L3/L4 or L4/L5	218	69.7 (47–88)	96/122
Kuo	2019	USA	Retrospective cohort	DLS with LSS	NA	601 (ULBD: 164; fusion: 437)	68.5 ± 9.6; 69.2 ± 9.6	59/105; 125/312
Kelly	2019	USA	Retrospective cohort	DLS	grade 1	119 (PLF: 49; PLF + TLIF: 70)	68 ± 10; 65 ± 10	22/27; 24/46
Chan	2019	USA	Retrospective cohort	DLS	grade 1	426 (decompression alone: 84; fusion: 342)	69.9 ± 10.5; 60.7 ± 11.0	43/41; 131/211
Chan (1)	2019	USA	Retrospective cohort	DLS	grade 1	143 (MIS TLIF: 72; MIS decompression: 71)	62.1 ± 10.6; 72.3 ± 9.7	32/40; 32/39
Vorhies	2018	USA	Retrospective cohort	DLS	NA	75024 (decompression: 6712; fusion: 68312)	69; 61	2776/3936; 25,645/42667
Veresciagina	2018	Switzerland	Prospective cohort	DLS	grade 1 and 2	36	66.53 (47–80)	6/30
Irmola	2018	Finland	Prospective cohort	DLS	NA	189	62 (24–87)	NA
Hayashi	2018	Japan	Retrospective cohort	DLS	grade 1 and 2	50 (CBT-PLIF: 20; MEL: 30)	69.3; 71.2	5/15; 7/23
Kato	2017	Japan	Prospective cohort	DLS	grade ≤ 1	51	70.8 ± 7.9	20/31
Gerling	2017	USA	Prospective cohort	DLS	NA	406 (292 underwent instrumented fusion, 85 non-instrumented fusion, and 29 decompression alone)	65.3	128/278
Cheung	2016	China	Retrospective cohort	DLS	grade 1	64	69.1 ± 8.7	29/36
Sato	2015	Japan	Retrospective cohort	DLS	NA	163 (decompression alone:74; decompression and fusion: 89)	65.8 ± 8.9	73/90
Macki	2015	USA	Retrospective cohort	DLS	grade 1 and 2	103 (PLF:58; PLF + PLIF/TLIF:45)	59.27 ± 11.35; 55.44 ± 10.18	26/32; 14/31
Blumenthal	2013	USA	Prospective cohort	DLS with LSS	grade 1	40	68.2 ± 7.7	10/30
Rihn	2012	USA	Retrospective cohort	DLS	NA	601	66	189/412
Booth	1999	USA	Retrospective cohort	DLS	NA	41	66.7 (52.2–78.7)	12/29

Author	BMI (kg/m^2^)	Disease duration (m)	Surgery types	Follow-up (years)	Number of reoperations	Reasons for reoperation	Reoperation methods	Risk factors of reoperation
Salimi	24.3 ± 3.9	36.2 ± 39.6	bilateral decompression	5	2	progression of lumbar degeneration or postoperative instability	NA	NA
Noh	NA	NA	OD followed by spinal fusion, laminectomy, and PELD	10	549; 33	NA	NA	NA
Moayeri	27.7 ± 4.2	NA	a unilateral laminotomy for ipsilateral decompression	8.2	22	NA	decompression (12), fusion (10)	NA
Liang	23.70 ± 3.5; 24.30 ± 2.9	< 6 mos:10; > 6 mos: 94	PMTD; MIS TLIF	2	3; 1	NA	NA	NA
Joelson	27.8 ± 5.3; 26.7 ± 4.0	NA	decompression and fusion; decompression only	7.6	2; 6	spinal stenosis or disk herniation at the index level (L3-4), the first cranial adjacent level (L2-3), and the first caudal adjacent level (L4-5)	NA	NA
Georgiou	NA	NA	anterior fusion; posterior fusion	2	2536; 8101	NA	NA	NA
Chan	30.7	NA	posterior lumbar spine decompression with or without single level posterior instrumented fusion	NA	438	NA	NA	age, sex, BMI, smoking, addition of fusion
Takaoka	NA	NA	single-level OLIF and single-level TLIF	3	3; 2	NA	NA	NA
Sugiura	NA	NA	BPL and PLIF	3	4; 4	root radiculopathy (4); ASD (3), left L5/S foraminal stenosis (1)	PLIF (3), BPL (1); PLIF (3), herniotomy (1)	NA
Rabah	30.85 ± 6.48	NA	posterior/transforaminal lumbar interbody fusion (P/TLIF)	NA	NA	NA	NA	operative time, obesity class III, non-insulin-dependent diabetes, smoking
Mimura	NA	NA	single-level unistrumented PLF	5	1	ASD	NA	NA
Katuch	25.56 ± 5.12; 23.78 ± 4.78	NA	TLIF and PLIF	3	2; 15	wound infection and dural tear	NA	NA
Joelson	27 ± 4.5; 27 ± 4.1	NA	decompression and fusion; decompression only	7.8	216; 80	spinal stenosis with or without concomitant DLS or disk herniation	NA	NA
Badhiwala	NA	NA	laminectomy alone; laminectomy plus fusion	5	18; 28	related to the principal procedure	NA	NA
Zhong	NA	NA	Posterolateral fusion; intervertebral fusion	4.2	24; 9	NA	NA	BMI, sex, diabetes mellitus, age, fusion method, more bleeding
Nyström	NA	NA	bilateral laminotomy	6.8	29	NA	decompression (24); fusion (5)	NA
Lee	NA	NA	decompressive laminectomy; PLIF; ALIF	10	52; 13; 1	occurrence of any lumbar spinal surgery with a disease code	NA	NA
Khan	30.96 ± 6.21	39.64	elective open posterior lumbar spinal fusion	1.9	31	NA	NA	diabetes
Khan	30.95 ± 5.82	NA	an open posterior lumbar fusion	1.3	7	NA	NA	NA
Karsy	NA	NA	percutaneous screw placement, interbody placement, or decompression	3	38	NA	NA	NA
Chan	29.5 ± 5.1; 31.3 ± 7.0	NA	minimally invasive or open TLIF	2	1; 16	ASD; surgical site infection; implant removal/revision; pseudoarthrosis	NA	NA
Bisson	28.735 ± 5.380; 28.190 ± 4.688	NA	MIS decompression or open decompression	3	10; 3	re-emergence of symptoms; persistent symptoms; recurrence of rt-sided pain	NA	NA
Minamide	NA	NA	micro-endoscopic decompression	2.3	17	NA	fusion (9); decompression (8)	NA
Kuo	NA	NA	ULBD or fusion	3.3	17; 75	NA	fusion; decompression; incision and drainage, revision instrumentation, instrumentation removal	fusion as index operation, BMI, any perioperative complication, estimated blood loss
Kelly	30.6 ± 5.9; 31.5 ± 6.7	NA	PLF or PLF + TLIF	2	8; 11	NA	NA	NA
Chan	28.4 ± 5.2; 31.0 ± 6.7	< 3 mos: 11; > 3 mos: 401	decompression alone or fusion	1	5; 15	wound revisions and/or incision and drainage, adjacent-segment disease, screw reposition/hardware failures, bony fracture within the fusion construct, and evacuation of hematoma	NA	NA
Chan (1)	29.5 ± 5.1; 28.2 ± 4.7	< 3 mos: 4; > 3 mos: 133	MIS TLIF or MIS decompression	2	1; 10	ASD	fusion (5); decompression (6)	NA
Vorhies	NA	NA	decompression or fusion	5	686; 8469	recurrent stenosis or progressive instability	fusion; decompression	NA
Veresciagina	NA	NA	laminotomy	10.78	5	NA	decompression	NA
Irmola	28.2 ± 4.3	NA	instrumented lumbar spine fusion	3.9	30	acute complication; early failure; adjacent segment pathology; late failure	NA	NA
Hayashi	23.7; 23.1	39.7; 41.5	CBT-PLIF or MEL	2	3; 5	a hematoma perioperatively; a vertebral fracture in the adjacent level; same-segment disease; insufficient decompression	kyphoplasty; adjacent_x005f segment decompression; additional decompression and fusion	NA
Kato	NA	NA	microsurgical bilateral decompression via a unilateral approach	2	2	exacerbation of disc degeneration	NA	NA
Gerling	29.2	at least 12 weeks	instrumented fusion, non-instrumented fusion, and decompression alone	8	60; 20; 9	progressive spondylolisthesis, recurrent stenosis, complications, or other	NA	age, gender, moderate or severe stenotic levels, predominant back pain, physical therapy, neurogenic claudication, leg pain bothersomeness scale
Cheung	NA	NA	decompression only	7.1	9	mechanical back pain; residual leg pain	further decompression(2); Fusion(6); epidural steroid injection(1)	NA
Sato	23.1 ± 2.6	NA	decompression alone or decompression and fusion	5	25; 13	adjacent segmental disease; same segmental disease; infection; implant related; hematoma	decompression and fusion (25); decompression-only (8); others (5)	NA
Macki	NA	NA	NA	5.5	17; 4	degenerative disease progression	NA	NA
Blumenthal	NA	NA	laminectomy	3.6	15	mechanical low back pain	NA	NA
Rihn	29.2	at least 12 weeks	posterior decompressive laminectomy	4	59	NA	NA	obesity
Booth	NA	NA	decompression, autogenous iliac crest bone grafting, intertransverse process fusion, and segmental (pedicle screw) instrumentation	6.5	5	recurrent stenosis at adjacent levels with transition syndrome	NA	NA

*Abbreviation: LSS* lumbar spinal stenosis, *DLS* degenerative lumbar spondylolisthesis, *PMTD* paraspinal mini-tubular lumbar decompression, *MIS TLIF* minimally invasive transforaminal lumbar interbody fusion, *OLIF* oblique lateral interbody fusion, *TLIF* transforaminal lumbar interbody fusion, *BPL* bilateral partial laminectomy, *PLIF* posterior lumbar interbody fusion, *ALIF* anterior lumbar interbody fusion, *PLF* posterolateral lumbar fusion, *CBT-PLIF* posterior lumbar interbody fusion with cortical bone trajectory, *MEL* microendoscopic laminotomy, *OD* open discectomy, *PELD* percutaneous endoscopic lumbar discectomy, *PMTD* paraspinal mini-tubular lumbar decompression, *ULBD* unilateral laminotomy for bilateral decompression, *ASD* adjacent segment disease, *BMI* body mass index, *NA* not available

### Overall results and subgroup analysis results of reoperation rate

**Fig. 2:**
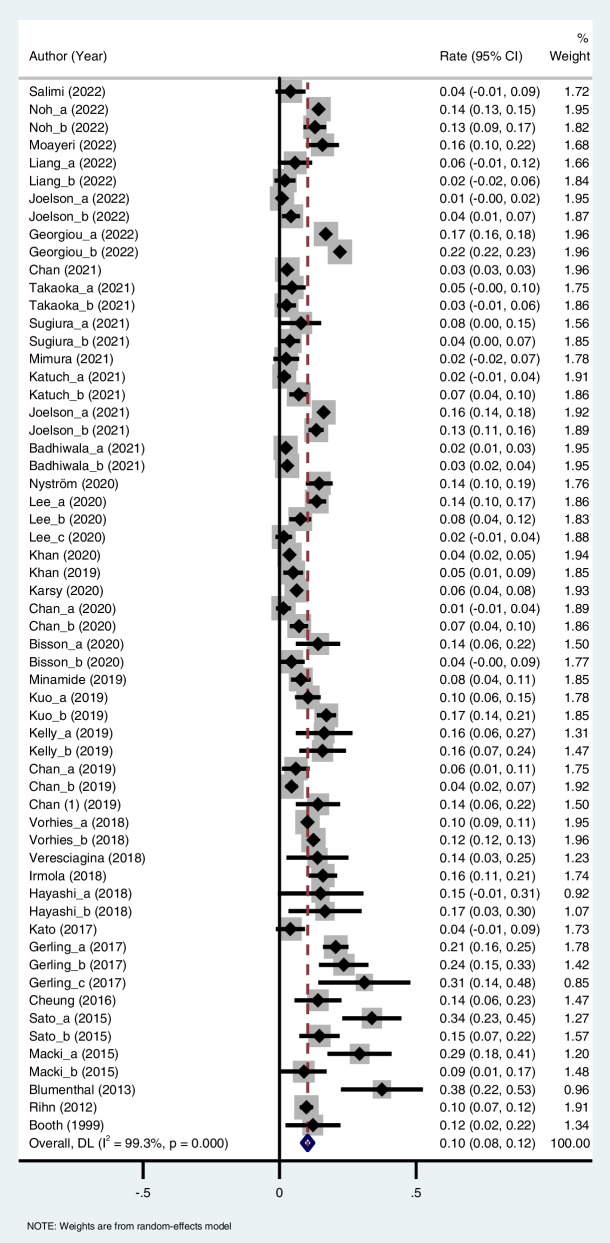
Forest plot regarding to reoperation rate

This meta-analysis showed a 10% (95%CI: 8%-12%) of reoperation rate in DLS patients (Fig. 2). A high heterogeneity was observed in the results (I^2^ = 99.3%). To explore the source of heterogeneity, subgroup analyses were performed. In study design subgroup, 10% of reoperation rate was found in both prospective cohort study (95%CI: 7%-13%) and retrospective cohort study (95%CI: 8%-13%). In patients subgroup, DLS patients showed 11% of reoperation rate (95%CI: 8%-13%) and DLS patients with LSS showed 10% of reoperation rate (95%CI: 6%-13%). In surgery types subgroup, the reoperation rate was 11% (95%CI: 9%-13%) in patients undergoing decompression, 10% (95%CI: 7%-12%) in patients undergoing fusion, 9% (95%CI: 5%-13%) in patients undergoing decompression and fusion, and 7% (95%CI: 3%-11%) in patients undergoing other surgeries. In follow-up time subgroup, the reoperation rate was 9% (95%CI: 6%-12%), 12% (95%CI: 9%-14%), and 10% (95%CI: 6%-15%) at follow-up time < 5 years, between 5 to 10 years, and ≥ 10 years, respectively. In study quality subgroup, there was 11% (95%CI: 9%-13%) of reoperation rate in studies with fair quality and 7% (95%CI: 5%-10%) of reoperation rate in studies with high quality. The overall and subgroup analysis results were shown in Table 2.

**Table 2:** Meta analysis of reoperation rate

Outcomes	Number of studies	Rate (95%CI)	I^2^
**Reoperation rate**	37	0.10 (0.08–0.12)	99.3
Sensitivity analysis		0.10 (0.08–0.12)	
Publication bias		Z = 2.91	*P* = 0.004
Study design			
Prospective cohort	12	0.10 (0.07–0.13)	94.5
Retrospective cohort	25	0.10 (0.08–0.13)	99.5
Patients			
DLS	27	0.11 (0.08–0.13)	99.4
DLS with LSS	10	0.10 (0.06–0.13)	96.5
Surgery types			
Decompression	21	0.11 (0.09–0.13)	91.9
Fusion	8	0.10 (0.07–0.12)	98.9
Decompression and fusion	6	0.09 (0.05–0.13)	99.0
Others	2	0.07 (0.03–0.11)	23.4
Follow-up (years)			
< 5	20	0.09 (0.06–0.12)	98.5
5–10	13	0.12 (0.09–0.14)	98.1
≥ 10	3	0.10 (0.06–0.15)	92.8
Quality			
Fair	26	0.11 (0.09–0.13)	98.6
High	11	0.07 (0.05–0.10)	96.7

*Abbreviation*: *CI* confidence interval, *I*^*2*^ I-squared, *DLS* degenerative lumbar spondylolisthesis, *LSS* lumbar spinal stenosis

### Meta-analysis of risk factors for reoperation

**Fig. 3:**
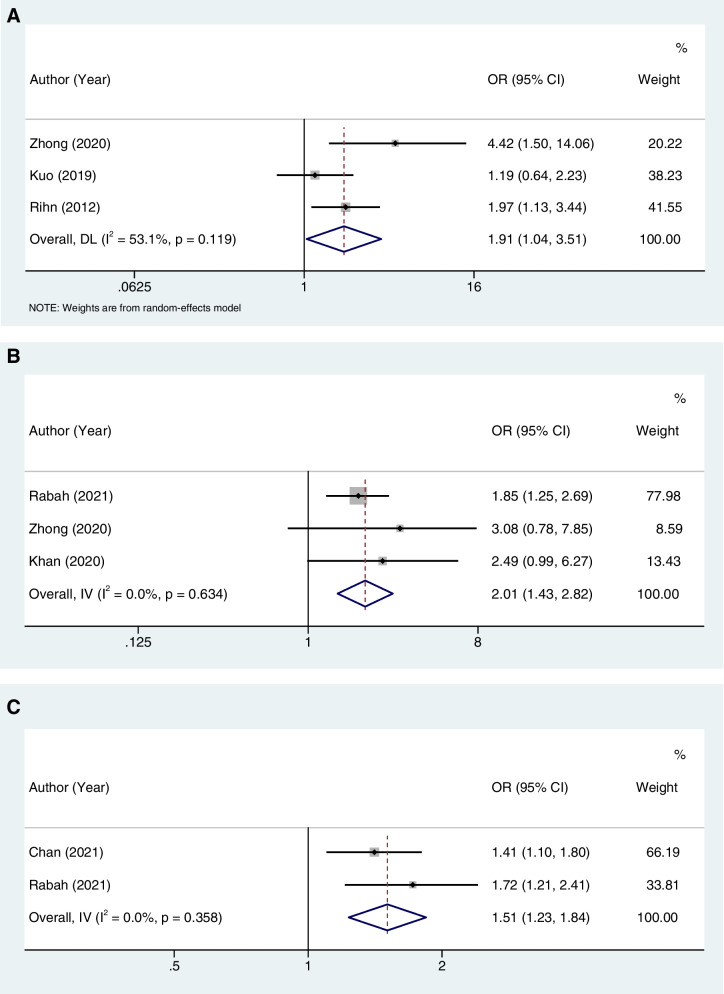
Forest plots regarding to obesity (**A**), diabetes (**B**), and smoking (**C**)

The meta-analysis showed that obesity (OR = 1.91, 95%CI: 1.04–3.51, I^2^ = 53.1%), diabetes (OR = 2.01, 95%CI: 1.43–2.82, I^2^ = 0%), and smoking (OR = 1.51, 95%CI: 1.23–1.84, I^2^ = 0%) were associated with an increased risk of reoperation. Age (OR = 0.99, 95%CI: 0.95–1.03, I^2^ = 78.4%), sex (OR = 1.31, 95%CI: 0.83–2.05, I^2^ = 60.4%), and more bleeding (OR = 0.86, 95%CI: 0.07–10.22, I^2^ = 87.5%) were not associated with the reoperation. The overall results were demonstrated in Table 3. Forest plots regarding to obesity, diabetes, and smoking were demonstrated in Fig. 3A, B, and C, respectively.

**Table 3:** Risk factors for the reoperation of DLS patients after surgeries

Risk factors	Number of studies	OR (95%CI)	*P*	I^2^
Age	3	0.99 (0.95–1.03)	0.535	78.4
Sensitivity analysis		0.99 (0.95–1.03)		
Sex	3	1.31 (0.83–2.05)	0.243	60.4
Sensitivity analysis		1.31 (0.83–2.05)		
Obesity	3	1.91 (1.04–3.51)	0.037	53.1
Sensitivity analysis		1.91 (1.04–3.51)		
Diabetes	3	2.01 (1.43–2.82)	< 0.001	0.0
Sensitivity analysis		2.01 (1.43–2.82)		
Smoking	2	1.51 (1.23–1.84)	< 0.001	0.0
Sensitivity analysis		1.51 (1.23–1.84)		
More bleeding	2	0.86 (0.07–10.22)	0.903	87.5
Sensitivity analysis		0.86 (0.07–10.22)		

*Abbreviation*: *DLS* degenerative lumbar spondylolisthesis, *OR* odds ratio, *CI* confidence interval, *I*^*2*^ I-squared

### Systematic review of risk factors for reoperation

This systematic review examined two literatures about the obesity. Chan et al. carried out a retrospective cohort study of obesity and reoperation after lumbar surgery [23]. As expected, significant higher risk of reoperation was found in patients who were obese [23]. Similar evidence was supported by Rabah et al. that an increase of one unit in BMI was associated with 4% increased risk of reoperation [11]. Moreover, study of Chan et al. showed addition of fusion was associated with higher risk of reoperation [23]. Rabah et al. found operative time > 5 h to be associated with an increased risk of reoperation [11]. A study consisted of 5-year follow-up indicated that having an index of fusion operation and perioperative complications was associated with the increased odds of reoperation [13]. Compared to intervertebral fusion, patients undergoing posterolateral fusion had 4.02-times risk of reoperation [8]. In addition, Gerling et al. have reported that patients with 2/3 moderate or severe stenotic levels, predominant back pain, no physical therapy, and greater leg pain score at baseline indicated higher reoperation rate [26].

### Assessment of publication bias and sensitivity analysis

Sensitivity analysis was performed by sequentially removing the study to assess the robustness of overall results. All results of sensitivity analysis were consistent with those of the main analysis (Tables 2 and 3). By funnel plot, we detected an evidence of publication biases (Z = 2.91, *P* = 0.004) (Table 2, Supplementary Fig. 1A). Therefore, a trim-and-fill method was utilized to fill the missing data to eliminate the impact of publication bias. Funnel plot with missing data filled was demonstrated in Supplementary Fig. 1B. Before filled, reoperation rate was 10% (95%CI: 8%-12%). After filled, reoperation rate was 11% (95%CI: 9%-13%).

## Discussion

The reoperation rate of DLS patients undergoing lumbar surgeries remains high in spite of improved surgical skills and techniques; therefore, exploring risk factors of reoperation is important [7, 8]. Considering the controversial results in the risk factors [8, 11–13], we performed a systematic review and meta-analysis based on currently available studies to analyze the reoperation rate and risk factors. In this study, we found a 10% of reoperation rate in DLS patients after lumbar surgeries. Obesity, diabetes, and smoking were identified as risk factors for the reoperation.

Several previous studies have reported the reoperation rate after lumbar surgeries in DLS patients [7, 49–52]. The reoperation rate was reported as 12.4% from 1990 to 1993 and 14.0% from 1997 to 2000 [51]. Ghogawala et al. proved that the reoperation rate was 15% at 1 year after the surgery in DLS patients only undergoing decompression [52]. In the present studies, the reoperation rate was found nearly the same as that reported in previous studies [7]. The reoperation rate in DLS patients was 15.7% at the mean follow-up of 8.2 years [7]. For patients undergoing fusion procedures, the cumulative reoperation rate was 14% [49]. Another report demonstrated that the reoperation rate ranged from 5.8% to 16.3% according to the type of surgeries [50]. Similar to the studies mentioned above, in our study, the reoperation rate of DLS was 10%, ranging from 8 to 12%. Our results may be useful for clinicians to evaluate the reoperation rate.

Identifying risk factors of reoperation for patients after lumbar surgeries is of clinical interest. In this study, obesity, diabetes, and smoking were found to be associated with higher risk of reoperation. Rabah et al. and Chan et al. have confirmed that smoking status was associated with greater risk of reoperation [11, 23]. Also, there were several studies reporting the positive association between obesity and reoperation of patients undergoing lumbar surgeries [8, 23]. This can be explained by that obese patients were more likely to be frail [53, 54], and frail patients had 56% increased odds of reoperation after lumbar surgery [23].

Animal studies have long recognized the close association between diabetes and lumbar spine disorders [55–57]. Diabetic models have revealed some harmful changes, such as increase of toxic end products of glycation, expression of matrix metalloproteinases 2 related to extracellular matrix degradation, and hyperglycemia-induced intervertebral disc inflammation, promoting intervertebral disc degeneration process [58–60]. Studies have revealed that diabetes was closely associated with degenerative lumbar spine disorders [61, 62]. Park et al. have found the influence of diabetes on the prevalence of lumbar spine surgeries, indicating that diabetes may be a factor aggravating lumbar spine disorders [62]. In Park et al. study, patients with diabetes underwent more lumbar surgeries than those without diabetes [62]. Their finding suggested that diabetes was significantly associated with the increased number of lumbar spine surgeries, and this finding is of critical importance because it revealed that diabetes may be an incentive for the increase of the severity of lumbar spine disorders, which ultimately led to the necessity of surgeries [62]. In this meta-analysis, diabetes was identified as a risk factor for the reoperation of DLS patients undergoing lumbar surgeries. This was consistent with the findings from Zhong et al. [8] Our findings suggested that when treating DLS patients with diabetes, physicians should pay more attention to glycemic control for the purpose of decreasing the risk of reoperation.

This meta-analysis explores the reoperation rate and risk factors for the reoperation. Results show that there is 10% of reoperation after lumbar surgeries, and obesity, diabetes, and smoking are found to increase the risk of reoperation. Our findings suggest that DLS patients should control glycemic level and weight, and reduce smoking to decrease the risk of reoperation. There are some limitations in this study. First, all fusion techniques (PLF and LIF) were put together. Due to the limitations of the included studies, it is unable to further analyze the reoperation rate in DLS patients undergoing the single fusion technique. Second, the number of studies reporting the risk factors of reoperation is relatively small, and some outcomes can only be qualitatively described, which may affect the stability of the results. Third, the risk of reoperation may be different according to the severity of lumbar spondylolisthesis and the first surgical methods; however, data provided in the currently available studies are insufficient to further analyze. Future meta-analysis including more relevant studies are needed to verify our findings and to explore the effect of lumbar spondylolisthesis severity and the first surgical methods on the risk of reoperation.

## Conclusion

Our meta-analysis found 10% of reoperation rate in DLS patients undergoing lumbar surgeries, and identified obesity, diabetes, and smoking as risk factors for the reoperation. Our findings suggested that patients should improve glycemic level and weight, and quit smoking to reduce the reoperation after lumbar surgery.

## Supplementary Information

**Additional file 1: Supplementary figure 1.** Funnel plot for publication bias before and after trim-and-fillanalysis.

## Data Availability

The datasets used and/or analyzed during the current study are available from the corresponding author on reasonable request.

## Abbreviations

DLS: Degenerative lumbar spondylolisthesis
PRISMA: Preferred Reporting Items for Systematic Reviews and Meta-Analyses
BMI: Body mass index
NOS: Newcastle-Ottawa Scale
OR: Odds ratio
CI: Confidence interval
