# Supplementary figures and images for: A systematic review and meta-analysis of risk factors for reoperation after degenerative lumbar spondylolisthesis surgery
Source: BMC Surg. 2023 Jul 5;23:192. doi: 10.1186/s12893-023-02082-8 (PMC10324215; doi:10.1186/s12893-023-02082-8)

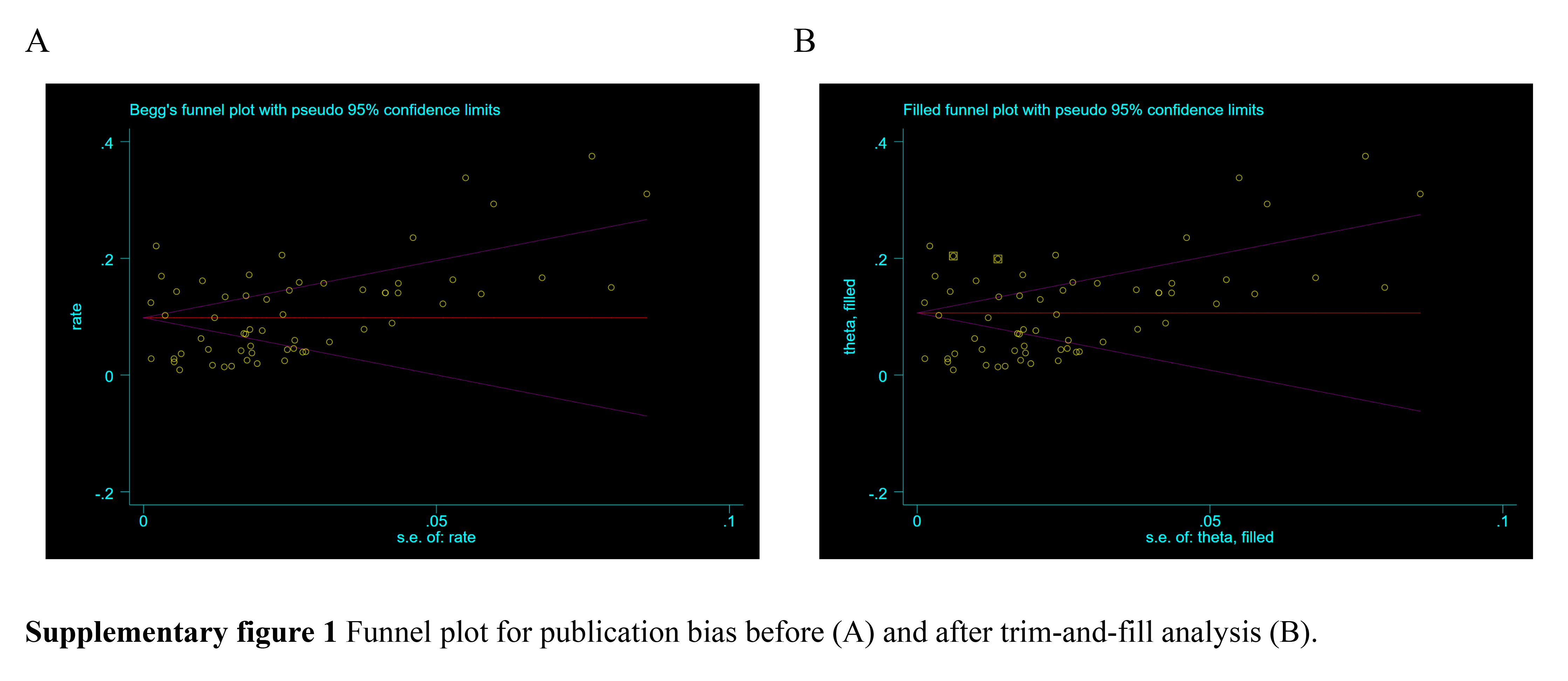

Supplement: Supplementary file 1 — Additional file 1: Supplementary figure 1. Funnel plot for publication bias before and after trim-and-fillanalysis. [file 12893_2023_2082_MOESM1_ESM.tif]
